# Severity of Dyskinesia and D3R Signaling Changes Induced by L-DOPA Treatment of Hemiparkinsonian Rats Are Features Inherent to the Treated Subjects

**DOI:** 10.3390/biom9090431

**Published:** 2019-09-01

**Authors:** Sacnité Albarrán-Bravo, José Arturo Ávalos-Fuentes, Hernán Cortés, Marina Rodriguez-Sánchez, Norberto Leyva-García, Claudia Rangel-Barajas, David Erlij, Benjamín Florán

**Affiliations:** 1 Departamento de Fisiología, Biofísica y Neurociencias, Centro de Investigación y de Estudios Avanzados del Instituto Politécnico Nacional, Ciudad de México 07360, Mexico; 2 Laboratorio de Medicina Genómica, Departamento de Genética, Instituto Nacional de Rehabilitación Luis Guillermo Ibarra Ibarra, Ciudad de México 14389, Mexico; 3 Neurosciences Program Bloomington, Indiana University, Bloomington, IN 47405, USA; 4 Department of Physiology, SUNY Downstate Medical Center, Brooklyn, NY 11203, USA

**Keywords:** dopamine 3 receptors, dopamine 1 receptors, parkinson’s disease, dyskinesia, basal ganglia, substantia nigra pars reticulata

## Abstract

Extensive damage to nigrostriatal dopaminergic neurons leads to Parkinson’s disease (PD). To date, the most effective treatment has been administration of levodopa (L-DOPA) to increase dopaminergic tone. This treatment leads to responses that vary widely among patients, from predominantly beneficial effects to the induction of disabling, abnormal movements (L-DOPA induced dyskinesia (LID)). Similarly, experimental studies have shown animals with widely different degrees of LID severity. In this study, unilateral injections of 6-hydroxydopamine (6-OHDA) in the medial forebrain bundle (MFB) produced more than 90% depletion of dopamine in both the striatum and the substantia nigra reticulata (SNr) of rats. Population analysis showed that dopamine depletion levels were clustered in a single population. In contrast, analysis of abnormal involuntary movements (AIMs) induced by L-DOPA treatment of 6-OHDA-lesioned animals yielded two populations: one with mild LID, and the other with severe LID, which are also related to different therapeutic responses. We examined whether the severity of LID correlated with changes in dopamine 3 receptor (D3R) signaling because of the following: (a) D3R expression and the induction of LID are strongly correlated; and (b) dopaminergic denervation induces a qualitative change in D3R signaling in the SNr. We found that the effects of D3R activation on cAMP accumulation and depolarization-induced [^3^H]-gamma-aminobutyric acid ([^3^H]-GABA) release were switched. L-DOPA treatment normalized the denervation-induced changes in animals with mild LID. The D3R activation caused depression of both dopamine 1 receptor (D1R)-induced increases in cAMP production and depolarization-induced [^3^H]-GABA release, which were reversed to their pre-denervation state. In animals with severe LID, none of the denervation-induced changes were reversed. The finding that in the absence of identifiable differences in 6-OHDA and L-DOPA treatment, two populations of animals with different D3R signaling and LIDs severity implies that mechanisms intrinsic to the treated subject determine the segregation.

## 1. Introduction

Because extensive damage to the dopamine-producing neurons of the substantia nigra pars compacta (SNc) causes Parkinson’s disease (PD), the preferred therapeutic strategies for this condition are based on substituting or increasing cerebral levels of the deficient neurotransmitter. In this regard, there is general agreement that the most effective treatment is the administration of the dopamine precursor levodopa (L-DOPA) [1]. Indeed, in the early stages of administration, L-DOPA therapy is quite successful; however, prolonged use leads to responses that range widely among patients, from predominantly beneficial effects to the induction of disabling, abnormal movements (L-DOPA induced dyskinesia (LID)) with distinct degrees of severity [2]. In experimental Parkinson’s disease, the generation of LID is also not uniform, since repeated injections of L-DOPA yield animals with different degrees of severity of LID [3,4,5,6,7,8,9,10,11,12]. Furthermore, in animals with different LID severity, neurochemical parameters change differently [3,4,6,7,8,12,13]. 

The speed of the development and intensity of LID is determined by external causes also observed in animal models (for a review, see Sharma et al. [14]), such as degree of damage to dopaminergic neurons [15], age at onset of PD [16], duration of treatment [16,17], dosage [15], and frequency of L-DOPA treatment [18]. In addition, it has been stressed that intrinsic genetic features of the parkinsonian subject may determine LID development [19]. 

In a previous study, population analysis of LID in hemiparkinsonian rats showed that the abnormalities were segregated into two populations: one with mild and the other with severe dyskinesia [11]. We hypothesize that further examination of these findings could test whether intrinsic factors comprise one of the major determinants of the intensity of LID. In particular, we determined whether: (a) our protocol of 6-hydroxydopamine (6-OHDA) treatment generates uniform lesions of dopaminergic structures and their relation to abnormal involuntary movements (AIM) scores [5,16,20]; (b) if populations with different AIM severity have distinct therapeutic response to L-DOPA treatment; and if (c) changes in dopamine 3 receptor (D3R) signaling are segmented in populations similar to those generated by AIM analysis. 

We examined D3R signaling in order to determine whether molecular changes correlate with the segmentation determined in the behavioral studies. We evaluated D3R responses because the expression level of the D3R in the striatum correlates with dyskinesia in mouse [21,22,23] and macaque monkey [24]. Indeed, it appears that D3R is the only dopamine receptor whose expression levels correlate linearly with LID severity [21,24,25]. Furthermore, overexpression of D3R in the rat striatum induces dyskinetic behaviors [26]. 

Also relevant is that during dopaminergic denervation, modulation by D3R of function of [^3^H]-gamma-aminobutyric acid (GABA) terminals in the substantia nigra pars reticulata (SNr) is qualitatively changed. Normally, activation of D3R enhances D1R signaling in the SNr and striatum [27,28,29,30]. However, after denervation, the effects of the activation of D3R are switched—instead of enhancing the response to D1R stimulation, D3R activation inhibits D1R responses [31]. Preliminary reports of these findings have been published [30].

## 2. Materials and Methods

### 2.1. The 6-OHDA Lesion and Animal Selection

The 6-OHDA lesion procedure was performed as described in our previous report [11]. In brief, Male Wistar rats were anaesthetized with ketamine/xilasine (75/5 mg/kg intraperitoneal; i.p.) and placed on a David Kopf stereotaxic frame and injected unilaterally with 6-OHDA (16 µg/1 µL of saline containing 0.1% ascorbic acid) in the medial forebrain bundle (MFB) at coordinates (A -1.8, L 2.4, V -7 mm) according to the atlas of Paxinos (1997). In order to prevent injury to noradrenergic neurons, animals were pre-treated with desipramine (10 mg/kg i.p.) 40 min prior to the surgical procedures. In order to ensure the degree of lesion, eight days after surgery animals were challenged with amphetamine (10 mg/kg i.p.) and tested for circling behavior. 

A set of 60 animals treated in this way were sacrificed and the striatal and nigral dopamine contents were determined. For studies of L-DOPA effects on AIMs, asymmetry index, and neurochemical determinations, rats showing 12 or more ipsilateral turns/minute at 30 min after injection were selected [11,32]. A set of 260 animals used in different projects of our lab were analyzed for AIMs, a set of 17 were used for determinations of AIMs and limb use asymmetry test, and a set of 17 were used for neurochemical determinations. All of the procedures were carried out in accordance with the National Institute of Health Guide for Care and Use of Laboratory Animals and were approved by the Institutional Animal Care Committee of the CINVESTAV (Protocol 0146-15; date of approval: November 30, 2015). 

### 2.2. Determination of Dopamine Content

Dopamine content in the striatum and SNr was determined as previously described [32]. One day after amphetamine challenge rats were decapitated, rat brains were removed, and 300 µM thick slices of SNr and striatum from the ipsilateral and contralateral side of the lesion were obtained by means of a vibratome. Slices were homogenized in 10 volumes of 0.1 M of HCIO_4_ containing 0.5 mM of sodium metabisulphite and centrifuged at 15,000× *g* for 30 s. The supernatant was filtered and dopamine content was determined by high performance liquid chromatography with electrochemical detection. Data were expressed as dopamine content in ng/mg protein. Percentage of depletion in striatum was calculated as the quotient of dopamine content in the lesioned side divided by the non-lesioned side and multiplied by 100. 

### 2.3. L-DOPA Treatment

Sixteen days after the 6-OHDA lesion, chronic L-DOPA treatment was initiated. L-DOPA methyl ester (10 mg/kg i.p.) plus benserazide (15 mg/kg i.p.) were given intraperitoneally [12,33,34]. After twenty days of L-DOPA treatment (i.e., 36 days after the original 6-OHDA lesion), animals were sacrificed to collect tissues for the [^3^H]cAMP accumulation assays and the experiments of [^3^H]-GABA release. 

### 2.4. Behavioral Observations

AIM tests rating was carried out as described by Cenci et al. [6]. The principles are similar to those applied in clinical dyskinesia rating scales [35]. Rats were observed individually for periods of 3 min every 20 min for three h following a daily dose of L-DOPA. The rating method assigns separate scores to three distinct topographical subtypes of abnormal movements (axial, limb, and orolingual AIMs, using a severity scale that is based on the proportion of time during which the observed behavior is present).

Movements were recognized as dyskinetic when the following criteria were met: (a) induced by L-DOPA; (b) only affected the contralateral side of the lesion; (c) were repetitive but not ascribable to any normal behavior pattern. For each of these 3 subtypes, each rat was scored on a scale from 0 to 4 based on the following criteria: 0 = not present; 1 = occasional (present during less than half of the observation time; 2 = frequent (present during more than half of the observation time); 3 = continuous (present all of the time but interrupted or suppressible by sensorial distraction); 4 = severity continuous (present all of the time but not suppressible by sensorial distraction). Thus, the maximum possible score in each session was 108 (where the maximum score per observation was 12 and the number of the observations per session was 9). We omitted the assessment of locomotive dyskinesia from the AIMs scores because changes in this parameter do not discriminate between animals with different degrees of dyskinesia [6,11,34,36]. Based on the data reported by Rangel et al. 2011 [11] and in the current paper, we classified all rats with AIMs scores above 32 as having severe dyskinesia, while all rats with a lower score were considered to have mild dyskinesia. This cutoff level is almost identical to that shown in Figure 1 of Fiorentini et al. [7] when adjusted for their shorter observation times. 

In order to measure the therapeutic effect of L-DOPA, we evaluated the limb-use asymmetry as an index of akinesia. Rats were placed in the cylinder according to the report of Schallert et al. [37] after the daily dose of L-DOPA on days 4, 9, and 19 of treatment. The number of wall contacts with the forelimbs ipsilateral and contralateral to the lesion side were counted over a period of 5 min every 20 min during the 180 min session. Limb use asymmetry was determined as the quotient of the number of contacts of the forelimb of the lesion side divided by the contacts of the forelimb of the non-lesioned side, assuming that if there is no akinesia in the lesion-side forelimb then indistinct wall contacts with the forelimbs will occur and the quotient will be near one. 

### 2.5. cAMP Accumulation Assay

The cAMP accumulation assays were performed as previously described by Alexander et al. [38]. Synaptosomal fractions were isolated from SNr slices. The slices were homogenized in buffer (sucrose, 0.32 M; HEPES, 0.005 M, pH 7.4), and then homogenates were centrifuged at 800× *g* for 10 min. The resulting supernatant was further centrifuged at 20,000× *g* for 20 min. From this second centrifugation the supernatant (S1) was discarded and the pellet (P1) was resuspended and collocated on the sucrose. Then, 0.8 M HEPES and 0.005 M buffer (pH 7.4) were again centrifuged at 20,000× *g* for 20 min. Finally, the supernatant was discarded and the new pellet (P2) containing synaptosomes was used. The fraction was incubated with [^3^H]-adenine (130 nM) for 1 h at 37 °C. After this period the fraction was suspended in Krebs-Henseleit buffer composition containing: NaCl, 127 mM; KCl, 3.73 mM; MgSO_4_,1.18 mM; KH_2_PO_4_, 1.18 mM; CaCl_2_, 1.8 mM; HEPES, 20 mM; Glucose, 11 mM; and 3-isobutyl-1-metylxantine 1 mM. Aliquots of 250 µL of the synaptosomes were placed in tubes and the drugs were added in 10 µL volumes. Incubation was continued for 15 min and stopped by adding 100µL ice-cold trichloroacetic acid (15%) containing unlabeled ATP (2.5 mM) and cAMP (4.5 mM). After a period of 20 min on ice, the tubes were centrifuged (4000 rpm, 5 min, 4 °C) and the supernatants loaded onto Dowex 50W-X4 (300 µL per column). A fraction containing [^3^H]-ATP was eluted with 3 mL of distilled water. A second eluent obtained with 5 mL distilled water was directly loaded onto neutral alumina columns. Alumina columns were finally eluted with 4 mL of 50 mM Tris-HCl buffer pH 7.4, to obtain [^3^H]cAMP. The results were expressed as ([^3^H]cAMP × 100)/([^3^H]cAMP + [^3^H]-ATP), and were normalized according to basal accumulation.

### 2.6. [^3^H]-GABA Release

The [^3^H]-GABA release was determined with methods described in detail by Nava-Asbell et al. [39]. Briefly, nigral slices collected from the lesioned and non-lesioned sides of 10 rats were pooled and left equilibrating for 30 min in artificial cerebrospinal fluid (aCSF) maintained at 37 °C and gassed with O_2_/CO_2_ (95:5 *v*/*v*), then they were incubated for 30 min in aCSF containing 80 nM [^3^H]-GABA (95 Ci/mmol). The labeling and perfusion solutions contained aminooxyacetic acid (10 μM) to prevent degradation of the label by GABA transaminase. At the end of this period, label excess was removed by washing twice with ice-cold aCSF that contained 100µM nipecotic acid to prevent recapture of [^3^H]-GABA. Nipecotic acid was also included in all solutions used in all of the following steps of the experiment. The slices were superfused with normal aCSF for 30 min before collecting fractions perfused with the aCSF at a flow rate of 0.5 mL/min for 30 min. Basal release of [^3^H]-GABA was measured by collecting 4 fractions of the superfusate (total volume 2 mL) before the slices were depolarized with a solution in which the [K^+^] was raised to 20 mM. Six more fractions were collected in the high K^+^ medium. All drugs were added to the medium in fraction 2; that is, before changing the superfusion to the high K^+^ medium, so as to explore effects on basal release. To determine the total amount of tritium remaining in the tissue at the end of the experiment, the slices were collected, treated with 1 mL of 1N HCl, and allowed to stand for 1 h before the scintillator was added.

The [^3^H]GABA release was expressed initially as a fraction of the total amount of tritium remaining in the tissue. The effect of drugs on the basal release of [^3^H]GABA was assessed by comparing the fractional release in fraction 2 (immediately before exposure of the tissue to drug) and fraction four (immediately prior to exposure to 20 mM of K^+^) using the paired t-test. Changes in K^+^-evoked [^3^H]-GABA release were assessed by comparing the area under the appropriate release curves between the first and last fractions collected after the change to high K^+^. 

### 2.7. Statistical Analysis

For population analysis, we plotted the percentage of depletion in striatum, turns/min, and AIMs scores for each rat and analyzed their frequency distribution. The bin width was calculated automatically with the GraphPad Prism 7.03 software (GraphPad Software, Inc., San Diego, CA, USA). The data were then adjusted to a single Gaussian and a sum of two-Gaussians, and the best fit was determined by the Akaike´s information criteria test (AICc). For the best fit we tested normality of residuals with the D’Agostino–Pearson, Shapiro–Wilk, and Kolmogorov–Smirnov normality test. The parameters for each population were calculated with the same software [11]. Linearity of the relationship between the percentage of depletion and amphetamine-induced turns was determined by linear regression and the significance of slope from non-zero was determined by F test. For the forelimb use asymmetry data, we compared the mean of asymmetry use to the theorical value of 1 by one sample t-test. 

The [^3^H]cAMP experiments were expressed as percent of control conditions and analyzed by one-way ANOVA followed by Dunett test for comparison with control or Tukey-Kramer multiple comparison test for comparison between several experimental conditions. In release experiments, the area under the release curve in the presence of elevated K^+^ was calculated for each individual chamber and averaged with the rest of the experimental group, then the data were analyzed by one-way ANOVA followed by Tukey-Kramer multiple comparison test. All analysis was performed using GraphPad Prism 7.03 software. 

### 2.8. Drugs

The following drugs were used: adenosine 3′,5′-cyclic monophosphate (cAMP); adenosine 5′-triphosphate disodium salt hydrate (ATP); 4′-acetyl-*N*-[4-[4-(2-methoxyphenyl)-1-piperazinyl]butyl]-[1,1′-biphenyl]-4- carboxamide (GR 103,691); amphetamine hydrochloride (amphetamine); *O*-(carboxymethyl) hydroxylamie hemihydrochloride (aminooxyacetic acid); desipramine hydrochloride (desipramine); l-3,4-dihydroxyphenylalanine methyl ester hydrochloride (L-DOPA methyl ester); (±)-β-homoproline (nipecotic acid); (+)-1-phenyl-2,3,4,5-tetrahydro-(1*H*)-3-benzazepine-7,8-diol hydrochloride (SKF38393); dl-Serine 2-(2,3,4-trihydroxybenzyl)hydrazide hydrochloride (benzeraside); (+)-(4aR,10bR)-3,4,4a,10b-Tetrahydro-4-propyl-2*H*,5*H*-[1]benzopyrano[4,3-b]-1,4-oxazin-9-ol hydrochloride (PD 128,907); 2,4,5-Trihydroxyphenethylamine hydrochloride, 6-hydroxydopamine (6-OH).

The following radiochemicals: Adenine, [2,8-3H]-, >97%, 1mCi (37MBq), [^3^H] adenine; aminobutyric acid (GABA) γ-[2,3-3H(N)]-, specific activity: 25-40Ci (925GBq-1.48TBq)/mmol, 1mCi (37MBq), [^3^H] GABA. All were purchased from Perkin Elmer.

## 3. Results

### 3.1. Analysis of L-DOPA- Induced AIMs Scores Yields Two Populations from a Single 6-OHDA-Lesioned Population

**Figure 1:**
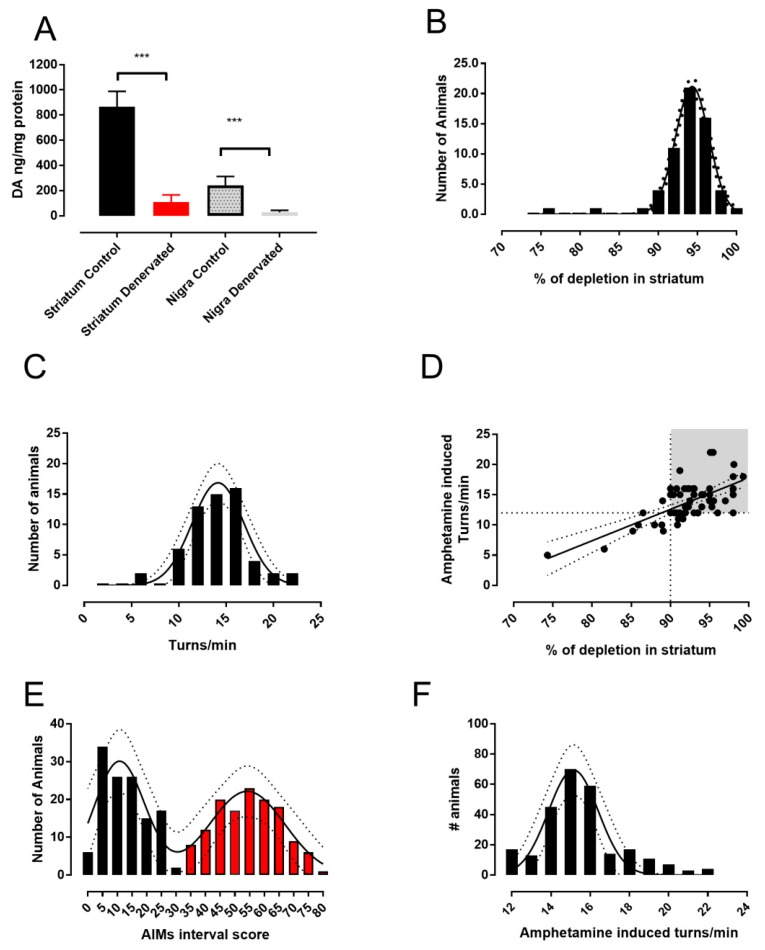
A single uniform 6-OHDA-lesioned population of rats produced two populations with low and high dyskinetic scores during L-DOPA treatment. (**A**) The effect is shown of 6-OH dopamine injected into the MFB on striatal and nigral dopamine content in the ipsilateral and the contralateral sides 17 days after the lesion (mean ± SD). (**B**) The population distribution of the percentage of striatal depletion. (**C**) The population distribution of this set of animals grouped by ipsilateral turns/min induced by a single dose of amphetamine (10 mgrs/kg i.p.) 16 days after the lesion. (**D**) The relationship of the percentage of dopamine depletion vs. amphetamine-induced circling of this set of animals. (**E**) The population distribution of a set of 260 animals selected for the amphetamine challenge with more than 12 turns/min and treated with L-DOPA 10 mgrs/kg i.p. over 20 days, grouped with their AIMs scores. Black bars indicate population one, corresponding to low scores, and red bars corresponding to the second population, corresponding to high scores. (**F**) The population distribution of this same set of 260 animals grouped by ipsilateral turns/min induced by a single dose of amphetamine (10 mgrs/kg i.p.) 16 days after the lesion. Note: ***p < 0.001 with respect to control side.

In a group of 60 denervated rats, we compared changes in dopamine content between control and 6-OHDA-treated sides to quantify the impact of the damage to dopaminergic structures in the striatum and the SNr after 21 days of lesioning with our method. As can be observed in Figure 1A, in the lesioned side of both structures, an important decrease in dopamine content with respect to the control side was found (*p* < 0.001). The degree of dopamine depletion was similar in both structures at nearly 90% in the mean.

Data from denervation were then analyzed for population distribution of the percentage of dopamine depletion using a non-linear fit to a single Gaussian distribution model and then compared with a fit to the sum of two Gaussian models.

Results indicated that data correspond better to a single population, since attempts to adjust the data to a sum of two Gaussian models showed ambiguous results. Comparison of fits using AICc indicated that the simpler model of a single Gaussian distribution has a higher probability of being the correct one (Figure 1B; best fit: population mean 94 ± 0.07, amplitude 21.16 ± 0.64, standard deviation (SD) 2.14 ± 0.07, *r*^2^ = 0.98. Normality tests: D’Agostino–Pearson K2 = 0.022, *p* = 0.98; Kolmogorov–Smirnov distance 0.199, *p* > 0.1, and Shapiro–Wilk W = 0.93, *p* = 0.34. Comparison of fits: difference in AICc -6.37, simpler model Gaussian probability 96.03%).

In order to test whether dopaminergic denervation produces two different populations in circling behavior induced by amphetamine [32], we also performed population analysis, as we did for dopamine depletion (Figure 1C). The results also indicated that data correspond much better to a single population (Best fit: population mean 14.14 ± 0.25, amplitude 16.85 ± 1.36, SD 2.74 ± 0.25, *r*^2^ = 0.94. Normality tests: D’Agostino–Pearson K2 = 0.332, *p* = 0.84; Kolmogorov–Smirnov distance 0.172, *p* > 0.1; Shapiro–Wilk W = 0.95, *p* = 0.70. Comparison of fits: difference in AICc −36.13, simpler model Gaussian probability 99%).

The linearity in the relationship between the degree of denervation measured by the percentage of depletion and amphetamine-induced turns was tested by linear regression fit (Figure 1D; slope 0.52 ± 0.07, *r*^2^ = 0.45, F = 47.47, df = 1.58, *p* < 0.001, F test). In all of our experiments, we used animals with 12 turns per min or higher, which corresponds to 90% depletion or higher, ensuring a homogenous degree of dopaminergic lesion [32,40].

Figure 1E plots the distribution of AIMs scores determined after treating hemiparkinsonian rats with L-DOPA for 20 days (*n* = 260, these data correspond to animals included in this paper and other projects from our laboratory). The results were best fitted by a sum of two Gaussians models (population one: mean 11 ± 1, amplitude 30 ± 4, and SD 8.6 ± 1.5; population two: mean 54 ± 2, amplitude 22 ± 3, and SD 13 ± 2, *r*^2^ = 0.88. Normality tests: D’Agostino–Pearson K2 = 3.99, *p* = 0.13; Kolmogorov–Smirnov distance 0.17, *p* > 0.1; and Shapiro–Wilk W = 0.93, *p* = 0.23). We also attempted to adjust the data to a simple Gaussian model; however, goodness of fit fell to an *r*^2^ of 0.16 and comparison of fits indicated a probability of 97.91% for the sum of two Gaussian models versus 2.09% for the simple Gaussian model. When we analyzed the models by population analysis and the rate of turning induced by amphetamine, the subjects behaved as if they were a single population (Figure 1F, population mean 15 ± 0.15, amplitude 67 ± 7, and SD 1.25 ± 0.15, *r*^2^ = 0.87. Normality tests: D’Agostino–Pearson K2 = 0.21, *p* = 0.9; Kolmogorov–Smirnov distance 0.147, *p* > 0.1; Shapiro–Wilk W = 0.95, *p* = 0.5). Comparison of fit versus a double-Gaussian model revealed a probability of 88.91% for the simpler model compared with 11.09% for the sum of two Gaussian models, indicating that the preferred model is the simple Gaussian one. Thus, the animals were nearly evenly divided between the two groups: severe dyskinesia had 134 animals, and mild dyskinesia had 126 animals.

### 3.2. Therapeutic Response to L-DOPA is Different in Mild and Severe Dyskinetic Animals

**Figure 2:**
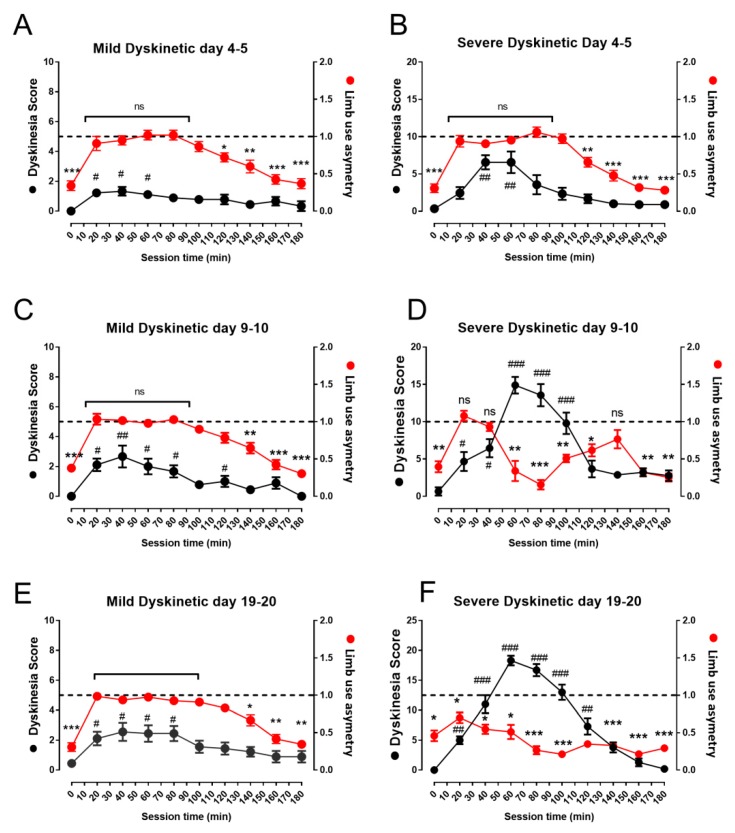
Comparison of the therapeutic response to L-DOPA measured by the limb asymmetry index and AIMs scores in mildly and severely dyskinetic animals classified at the end of L-DOPA treatment. (**A**,**C**,**E**) Graphs show the temporal course of the limb asymmetry index (right axes) and AIMs scores (left axes) during two continuous behavioral (180-min) evaluation sessions of a single dose of L-DOPA in mildly dyskinetic rats. (**B**,**D**,**F**) The effect on these same days in a set of severely dyskinetic rats. Data represent mean ± standard error of eight mildly dyskinetic vs. nine severely dyskinetic animals. Note: ns = significant differences, * *p* < 0.05, ** *p* < 0.01, and *** *p* < 0.001, between the value of the asymmetry index compared to 1; # *p* < 0.05, ## *p* < 0.01, and ### *p* < 0.001, with respect to initial score of dyskinesia.

In order to explore whether dyskinesia is related with differences in the therapeutic response to L-DOPA, we analyzed the temporal course of the effect of a dose of L-DOPA (10 mg/kg i.p.) in the limb asymmetry index, i.e., the therapeutic effect (in behavioral session at days 4, 9, and 19) and in AIMs score, i.e., the dyskinetic effect (in behavioral session at days 5, 10, and 20). A set of 17 lesioned rats previously selected by amphetamine challenge were separated into mild and severe dyskinetic at the end of L-DOPA treatment and data from temporal course of asymmetry index and AIMs scores were compared in a contiguous session day (i.e., day 4 for asymmetry index vs. day 5 for AIMs score, since measurements were taken in different behavioral boxes). In Figure 2A,C,E, we are able to observe that in animals that exhibited mild dyskinetic scores during the 180-min session (no more than 30 in the total session score; black circles, left axes of Figure 2A,C,E), they also showed a recovery in the limb-use asymmetry index from 20 to 100 min (red circles, right axes of Figure 2A,C,E). This behavior was observed on days 4, 9, and 19.

In animals that developed severe dyskinesia (more than 30 in the total session score; black circles, left axes of Figure 2B,D,F) at the end of the L-DOPA treatment, it can be observed that at the beginning of the treatment (day 5), the animals present the therapeutic window (Figure 2B), while dyskinetic scores remain below 10. Nevertheless, the therapeutic effect is lost when the dyskinesia score increases. In fact, a loss of therapeutic response is observed when dyskinesia reaches scores above 10 and is more evident on days 10 and 20 (Figure 2D,F).

Interestingly, dyskinesia scores remained constant during the 180 min session in animals with mild dyskinesia, but in animals classified as severely dyskinetic, a peak value in the scores is observed nearly 60 min after the injection. In addition, total session scores remained constant along the different treatment days in mild dyskinetic animals, while the scores increased with a maximal value near day 10 in severe dyskinetic animals (Figure 2G).

### 3.3. Denervation-Induced Changes in [^3^H]cAMP Accumulation by D3R Were Reverted in L-DOPA-Treated Animals with Mild Dyskinesia, but These Persist in Animals with Severe Dyskinesia

**Figure 3:**
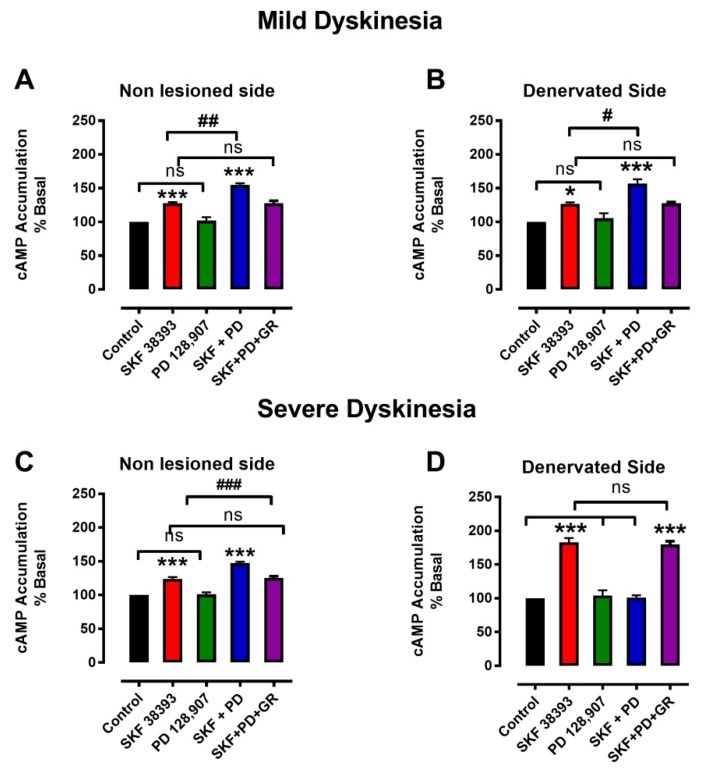
Changes in cAMP accumulation in the SNr of animals that developed either mild or severe dyskinesia during L-DOPA treatment. (**A**,**B**) Synaptosomes obtained from animals in which L-DOPA treatment produced mild dyskinesia. (**C**,**D**) Synaptosomes obtained from animals in which L-DOPA treatment produced severe dyskinesia. Note: *** *p* < 0.001, comparison with the control; # *p* < 0.05, ## *p* < 0.01, and ### *p* < 0.001, comparison with SKF38393; ns = not significant between groups. Values mean ± standard error, *n* = 3 experiments, four replications per experiment.

The effects of selective dopamine D1R agonist SKF38393 (1 μM) and D3R agonist PD 128,907 (100 nM) on [^3^H]cAMP accumulation in nigral synaptosomes of animals pretreated with L-DOPA over 20 days are presented in Figure 3. The doses of those drugs were chosen on the basis of dose-response and affinities calculated in previous reports of our work group [11,31,39,41]. It is timely to mention that those doses are in a suitable range of selectivity for their receptors. In rats in which L-DOPA treatment induced mild dyskinesia, the effects of co-treatment with SKF38393 and PD 128,907 in synaptosomes obtained from the lesioned and the non-lesioned side were nearly identical (Figure 3A,B). SKF38393 stimulated [^3^H]cAMP production and co administration of PD 128,907 further enhanced the stimulation of [^3^H]cAMP accumulation (*p* < 0.01 in the non-lesioned side; *p* < 0.05 in the lesioned side). The enhancing effect produced by PD 128,907 was prevented by D3R selective antagonist GR 103,691 (10 nM).

On the other hand, in animals that developed severe dyskinesia, the stimulation caused by SKF38393 alone was significantly higher in the denervated side than in the non-lesioned side ([^3^H]cAMP accumulation above that of the control, 24 ± 3% in the non-lesioned side vs. 83 ±3% in lesioned side, *p* < 0.001; Figure 3C,D). The stimulation caused by SKF38393 was also higher than that reported by Ávalos-Fuentes et al. [31] in the denervated side of 6-OHDA-denervated rats ([^3^H]cAMP accumulation above that of the control: 83.3 ± 3% vs. 62 ± 2%).

Furthermore, co-treatment with D3R agonist PD 128,907 inhibited the stimulatory effect of SKF38393 on the lesioned side (Figure 3D). The inhibitory effect produced by PD 128,907 on D1R stimulation was prevented by D3R selective antagonist GR 103,691 (Figure 3D). These responses are similar to those previously observed in the denervated side of animals that were not treated with L-DOPA [31].

In the non-lesioned side of slices obtained from animals with severe dyskinesia, SKF38383 significantly stimulated [^3^H]cAMP accumulation (*p* < 0.001) and the subsequent co-treatment with PD 128,907 further increased [^3^H]cAMP accumulation (Figure 3C). The enhancement effect produced by PD 128,907 was prevented by D3R selective antagonist (Figure 3D).

### 3.4. Denervation-Induced Changes in K^+^-Stimulated [^3^H]-GABA Release Were Reverted in L-DOPA- Treated Animals with Mild Dyskinesia, but These Persist in Animals with Severe Dyskinesia

**Figure 4:**
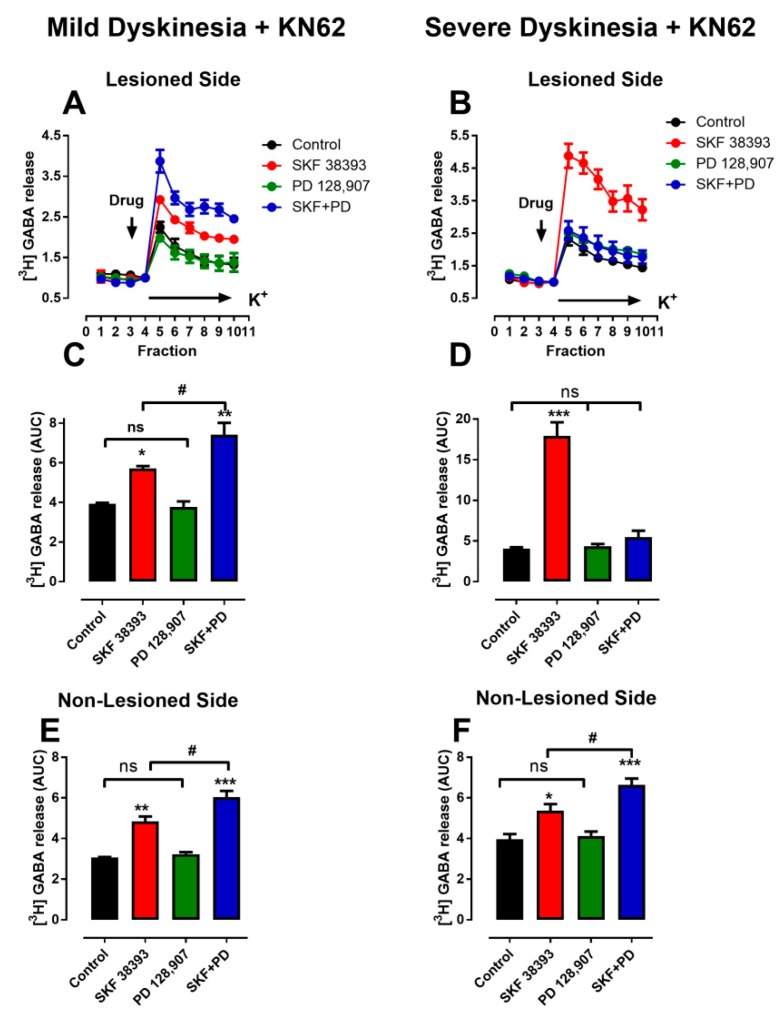
Effects of D3R activation on D1R stimulation on K^+^-depolarization-induced [^3^H]-GABA release in SNr slices. (**A**,**B**) Temporal courses of K^+^-depolarization-induced responses. Vertical arrow indicates time when ligand perfusion was initiated; horizontal bar indicates initiation of perfusion with 20 mM K^+^ solution. (**C**,**D**) Results for lesioned side of animals with mild dyskinesia and severe dyskinesia, respectively. (**E**,**F**) Non-lesioned side of animals with mild dyskinesia and severe dyskinesia, respectively. Note: # *p* < 0.05, * *p* < 0.05, ** *p* < 0.01, and *** *p* < 0.001 in comparison with control; ns is not significant. Values mean ± standard error, *n* = 3 experiments, five replications per experiment.

The responses of K^+^-stimulated [^3^H]-GABA release determined in slices of the SNr from 6-OHDA- lesioned and non-lesioned sides of animals that were pre-treated with L-DOPA over 20 days are depicted in Figure 4.

Previously, we demonstrated that the stimulatory effects of D3R activation on [^3^H]-GABA release were absent during K^+^-depolarization in non-lesioned tissues, in a process mediated by the phosphorylation-dependent activation of CaMKIIα [41]. Therefore, in order to study only the role of D3R on GABA release, the CaMKIIα-mediated inhibition of D3R action was prevented by selective inhibitor KN-62 (4µM) [41] in this set of experiments.

In the non-lesioned side of animals with either mild (4E) or severe dyskinesia (4F), stimulation of D3R with PD 128,907 enhanced the effects of D1R agonist SKF38393 to a similar degree. Likewise, in the lesioned side of animals with mild dyskinesia, stimulation of D3R with PD 128,907 also enhanced the effects of D1R agonist SKF38393 (Figure 4C). However, in the denervated side of animals with severe dyskinesia, the administration of D3R agonist PD 128,907 prevented the stimulatory effects of activation of D1R with SKF38393 (Figure 4D). Comparison of Figure 4C and Figure 4D revealed that in animals with severe dyskinesia, SKF38393 effects were significantly greater (*p* < 0.001) in slices obtained from the lesioned side (17.94 ± 1.65) than in slices from the non-lesioned side (4.84 ± 0.20). The effect was also greater than the response reported by Ávalos-Fuentes et al. [31] in their denervated tissues without L-DOPA treatment (12.21 ± 1.26). This pattern of D3R action in severely dyskinetic animals is similar to that observed in our previous experiments in which denervated animals were not treated with L-DOPA [31].

## 4. Discussion

### 4.1. Separate LID Populations of Rats with the Same Degree of Lesion

Our findings demonstrated that by utilizing a uniform lesion and treatment protocol in hemiparkinsonian rats, L-DOPA treatment yielded two populations: one with mild, and the other with severe dyskinesia (Figure 1E).

In our model, the uniformity of the 6-OHDA-induced lesions was evaluated by determining dopamine depletion and amphetamine-induced turning (Figure 1D). In agreement with prior studies [15,32,42,43,44,45], the rate of turns induced by amphetamine and the level of dopaminergic cell damage were linearly related, which permitted the selection of animals with the highest degrees of lesions (more than 90%; Figure 1D, shadow). Also, population analysis of both parameters turning behavior and dopamine depletion yielded a single population (Figure 1B,C).

Although the variability in the severity of LID has been previously recognized both in patients as well as in experimental studies [2,46,47], the remarkable feature of our observations is that the results are fitted by the sum of two Gaussian models, indicating the presence of two different populations and suggesting an approximate objective value where they can be separated. Since the qualitative differences between the two populations are generated by the same treatment schedule, it is very likely that the difference in LID severity is linked to the different expression of molecular mechanisms rather than to differences in the treatment protocol, which is in concordance with previous studies [48]. In fact, the changes in the effects of D3R on cAMP accumulation and GABA release observed in denervation [31] continued and were enhanced in animals that developed severe dyskinesia, supporting the idea that previously initiated molecular changes are preserved and enhanced only in these animals (Figure 3D and Figure 4D). Thus, we think that LID is also related to intrinsic factors that are possibly genetic [14]. Remarkably, this notion is supported by very recent studies that have demonstrated genetic features as major factors influencing susceptibility to dyskinesias in patients with Parkinson´s disease [49,50].

### 4.2. LID and Therapeutic Response to L-DOPA

Our data also revealed a different therapeutic response to L-DOPA related to animals with mild dyskinesia and with severe dyskinesia; the rats with severe dyskinesia lost the recovery of limb use near day 10 of treatment, whereas mildly dyskinetic rats retained it. This indicates that animals with mild dyskinetic scores have a therapeutic window in response to L-DOPA treatment that remains constant throughout the treatment, as occurs in patients that do not develop dyskinesia [51], whereas in animals with severe dyskinetic scores, this window is lost as treatment continues and LID scores continue to increase. In this respect, it has been shown that parkinsonian symptoms are present during peak-dose LID [52,53,54,55]. This also occurs in the animal model employed herein, suggesting that loss of therapeutic effect comprises another feature of the differences between animals with mild and severe dyskinesia.

In another study [7], two populations of L-DOPA-treated animals were also identified by evaluation of the therapeutic effects of L-DOPA on the unilateral akinesia of 6-OHDA-lesioned rats and the AIMs scores induced by L-DOPA. These criteria related the therapeutic response measured by the loss of the recovery of limb-use asymmetry to high dyskinetic scores and vice versa, i.e., improvement of limb-use asymmetry with low dyskinetic scores. In our study, AIMs scoring also identified two populations of L-DOPA-treated rats, which were remarkably similar to those described by Fiorentini et al. [7]. The scores for determining non-dyskinetic and dyskinetic animals were also similar to ours when adjusted to shorter observation periods (120 min vs. 180 min in this study). Moreover, the dose of L-DOPA employed was the same (10 mgrs/kg) and the relative proportion of non-dyskinetic animals found was approximately 39% (corresponding to our mildly dyskinetic group) vs. 46% in our study. This indicates that both assays with similar degrees of lesion (95% vs. 94%) and a similar dose of L-DOPA can separate the two groups. Thus, loss of therapeutic effect associated with high dyskinetic scores and vice versa might represent the expression of intrinsic factors related to L-DOPA therapy. In support of this hypothesis, other studies employing different measurements of dopaminergic damage of more than 90% found severe and eukinetic, non-dyskinetic, or mildly dyskinetic animals at similar proportions with similar protocols of treatment [5,6,11,12,56,57]. Therefore, our study demonstrated for the first time (to our knowledge) that these groups represent different populations of subjects.

### 4.3. Changes in cAMP as Determinant of LID

In its normal state, the D3R potentiates the D1R effect on GABA release and cAMP in striato-nigral neurons [29]; during denervation, this interaction becomes antagonistic [31], and it is supposed that L-DOPA treatment should restore it. However, in our study, this only occurred in mildly dyskinetic animals, whereas in severely dyskinetic animals, the antagonist interaction was enhanced and unexpected.

Many neurochemical abnormalities have been identified during LID generation [2]. Perhaps the most relevant of these in terms of the present findings are the changes in the D1R–cAMP–PKA signaling pathway, because D3R signaling is dependent on interactions with D1R. LID are associated with increased recruitment of D1R at the cell surface [58] and with overexpression of adenylyl cyclase type 5 (ACV) in neurons of the direct pathway [11,59]. In the present study and in our previous report [11], the increase in D1R-stimulated cAMP production in denervated animals with severe dyskinesia was above that produced in animals with mild dyskinesia. This additional signaling through the D1R–cAMP–PKA signaling pathway may be a major underlying abnormality involved in LID generation [56], in that it is related with GABA release.

Regarding this point, Sancesario et al. [12] measured extracellular cAMP levels and cGMP during the active phase of dyskinesia, finding a lower extracellular and intracellular content of these messengers in striatum after L-DOPA administration, contrariwise to the idea that an increase in cAMP production related to D1R receptor supersensitivity and GABA release takes place during dyskinesia. Different experimental conditions could be responsible for these differences. Perhaps the most important of these is that in our study observations were performed 1 day after the last dose of L-DOPA, and in the study by Sancesario et al. was during L-DOPA effect, and of course, also in vivo. Another possible explanation for this discrepancy is the effect of D3R on ACV activity stimulated by D1R. Because L-DOPA can activate both types of receptors, it is possible that the increased cAMP production stimulated by the D1R could be counteracted by D3R, generating a decreased cAMP production and content, as occurred in our experiments. This idea is possible, since the effect of D3R on D1R signaling occurs during ACV signaling [31]. In addition, the findings of Sancesario et al. [12] indicate that the decreased cAMP is related to a greater extent with a decrease in synthesis rather than with increased catabolism, which is compatible with the canonical antagonistic interaction fingerprint of D1R/D3R heteromers in cAMP production [60]. It should be noted that these heteromers are overexpressed in dyskinetic animals [61]. Thus, during the development of dyskinesia, it would be feasible to measure low levels of cAMP. This change in second messenger, together with changes in the expression of protein related to its synthesis, such as ACV [11], are some of the intrinsic factors related with the expression of dyskinesia in animals.

### 4.4. D3R Changes and LIDs

The two LID populations were also correlated with different responses in D3R signaling. The changes in GABA release induced by D3R activation were very different in the two groups (Figure 4). Activation of D3R in the SNr was transformed during denervation from an “atypical” potentiating response into a “typical” inhibitory response [41,62,63], while L-DOPA treatment was able to reverse this change only in the animals with mild dyskinesia. Thus, in L-DOPA-treated animals with severe dyskinesia, activation of D3R inhibited the stimulation caused by D1R activation (Figure 4D). This drastic change in response suggests that it may be a well-suited marker for assaying the underlying molecular changes induced by denervation and L-DOPA. This typical signaling of D3R on GABA release is a conflicting result when attempting to explain motor behavior and dyskinesia. These are exaggerated, uncontrolled movements that could be better understood if we assumed the atypical signaling.

For quite some time it has been widely accepted that LID are generated by depressed neuronal firing in the main output structures of the basal ganglia (GPi and SNr), leading to increased impulse traffic across thalamic relay nuclei [64,65]. In this regard, both in vitro studies [11,66] and in vivo microdialysis determinations [34,67] have shown that LID are consistently associated with increased L-DOPA-induced GABA release in the SNr, which is blocked by D1R antagonism. Our present findings demonstrate that D3R stimulation depresses the GABA release induced by D1R stimulation in SNr when severe dyskinesia is present. This reduced GABA release is expected to increase the firing of SNr neurons and to depress the relay nuclei of the thalamus, thus inhibiting movement and LID [68,69]. Namely, our findings imply that D3R activation in GABAergic terminals of the SNr of animals with severe LID ought exert an antidyskinetic effect. In agreement with this view, it has been observed that D3R agonists depress LID [24,70,71]. However, other reports have shown that D3R antagonists [71,72,73,74] and the deletion of D3R [75] also reduce LID. The only discrepant finding is found in the report of Mela et al. [76], revealing that antagonist S33084 did not diminish LID. Although some of the inconsistencies may be partially explained by differences in experimental design and the lack of optimal specificity of the D3R ligands tested, the effects of deleting D3R are particularly persuasive [75]. Therefore, other circuits or neurochemical changes may be also critically involved in the process. For example, D3R are also expressed in subthalamic projections to the SNr [77], where they depress transmitter release [78,79,80]. This subthalamic-nigral pathway could play a major role in the generation and management of LID, because the blockade of D3R would increase glutamate release that, in turn, would activate ionotropic receptors in the cell bodies and dendrites of SNr neurons, increasing their firing rate [68]. This increased firing rate would depress motor activity, an effect that would be in agreement with the antidyskinetic effect of D3R antagonists [71,72,73,74].

On the other hand, Mela et al. [34] found that the antagonism of mGluR5 has strong anti-dyskinetic effects in animals with severe LID and that it also depresses the increased GABA levels observed within the SNr of these animals [34,67]. Both the mRNA and protein for mGluR5 are present in the striatal GABAergic terminals of the SNr [81,82,83,84], and direct control of GABA release by activating these receptors has been demonstrated [85]. During LID, both glutamate and GABA levels are elevated in the SNr [67]. Therefore, the increased GABA levels found within the SNr during severe dyskinesia could be due, at least in part, to the increased activation of mGluR5 produced by the high levels of glutamate present in the SNr during LID generation [67]. If the glutamate effects on mGlu5Rs of GABAergic projections were dominant during LID, blockade of D3R would increase glutamate release, promoting GABA release via mGluR5 activation, thus increasing the generation of LID instead of opposing them. In this respect, genetic knock-down of mGluR5 mRNA in striato-nigral neurons prevents the development of dyskinesia [86], indicating the feasibility of this type of mechanism.

Finally, recent behavioral observations suggested that the generation of LID is related with the activation of D1R and D3R independently, and that co-activation potentiates their effect on LID, indicating that different kinds of interactions between D1R and D3R can occur at the signaling level, which could be related to dyskinesia [87]. ERK signaling could play an important role, since D1R and DR3 synergize and potentiate the signaling through this kinase [58] and are overexpressed in dyskinetic animals [88]. To our point of view in this discussion, several circuits, receptors, or receptor interactions could be responsible for the enhanced release of GABA, which is closely related with dyskinesia, and not only D3R. It is evident that such possibilities should be explored and more research is necessary to render them compatible or discard them. However, changes in D3R signaling are also an inherent factor related to LID.

## 5. Conclusions

Statistical analysis validated the existence of two populations of dyskinetic animals: one with mild and one with severe dyskinesia deriving from a single uniform lesioned population. Differences in the L-DOPA therapeutic response and D3R signaling in SNr (cAMP accumulation and GABA release) induced by dopaminergic denervation and L-DOPA treatment corresponded to the two populations identified by AIMs scoring. The qualitative difference responses suggest that these are produced by the expression of different molecular intrinsic factors.

## References

[1] Obeso J.A., Rodriguez-Oroz M.C., Goetz C.G., Marin C., Kordower J.H., Rodriguez M., Hirsch E.C., Farrer M., Schapira A.H., Halliday G. (2010). Missing pieces in the Parkinson’s disease puzzle. Nat. Med..

[2] Bastide M.F., Meissner W.G., Picconi B., Fasano S., Fernagut P.O., Feyder M., Francardo V., Alcacer C., Ding Y., Brambilla R. (2015). Pathophysiology of L-dopa-induced motor and non-motor complications in Parkinson’s disease. Prog. Neurobiol..

[3] Alcacer C., Santini E., Valjent E., Gaven F., Girault J.A., Herve D. (2012). Galpha(olf) mutation allows parsing the role of cAMP-dependent and extracellular signal-regulated kinase-dependent signaling in L-3,4-dihydroxyphenylalanine-induced dyskinesia. J. Neurosci..

[4] Bastide M.F., Dovero S., Charron G., Porras G., Gross C.E., Fernagut P.O., Bezard E. (2014). Immediate-early gene expression in structures outside the basal ganglia is associated to l-DOPA-induced dyskinesia. Neurobiol. Dis..

[5] Carta M., Lindgren H.S., Lundblad M., Stancampiano R., Fadda F., Cenci M.A. (2006). Role of striatal L-DOPA in the production of dyskinesia in 6-hydroxydopamine lesioned rats. J. Neurochem..

[6] Cenci M.A., Lee C.S., Bjorklund A. (1998). L-DOPA-induced dyskinesia in the rat is associated with striatal overexpression of prodynorphin- and glutamic acid decarboxylase mRNA. Eur. J. Neurosci..

[7] Fiorentini C., Rizzetti M.C., Busi C., Bontempi S., Collo G., Spano P., Missale C. (2006). Loss of synaptic D1 dopamine/N-methyl-d-aspartate glutamate receptor complexes in L-DOPA-induced dyskinesia in the rat. Mol. Pharmacol..

[8] Fiorentini C., Savoia P., Savoldi D., Barbon A., Missale C. (2013). Persistent activation of the D1R/Shp-2/Erk1/2 pathway in l-DOPA-induced dyskinesia in the 6-hydroxy-dopamine rat model of Parkinson’s disease. Neurobiol. Dis..

[9] Gardoni F., Picconi B., Ghiglieri V., Polli F., Bagetta V., Bernardi G., Cattabeni F., Di Luca M., Calabresi P. (2006). A critical interaction between NR2B and MAGUK in L-DOPA induced dyskinesia. J. Neurosci..

[10] Munoz A., Li Q., Gardoni F., Marcello E., Qin C., Carlsson T., Kirik D., Di Luca M., Bjorklund A., Bezard E. (2008). Combined 5-HT1A and 5-HT1B receptor agonists for the treatment of L-DOPA-induced dyskinesia. Brain.

[11] Rangel-Barajas C., Silva I., Lopez-Santiago L.M., Aceves J., Erlij D., Floran B. (2011). L-DOPA-induced dyskinesia in hemiparkinsonian rats is associated with up-regulation of adenylyl cyclase type V/VI and increased GABA release in the substantia nigra reticulata. Neurobiol. Dis..

[12] Sancesario G., Morrone L.A., D’Angelo V., Castelli V., Ferrazzoli D., Sica F., Martorana A., Sorge R., Cavaliere F., Bernardi G. (2014). Levodopa-induced dyskinesias are associated with transient down-regulation of cAMP and cGMP in the caudate-putamen of hemiparkinsonian rats: Reduced synthesis or increased catabolism?. Neurochem. Int..

[13] Fiorentini C., Savoia P., Savoldi D., Bono F., Busi C., Barbon A., Missale C. (2016). Shp-2 knockdown prevents l-dopa-induced dyskinesia in a rat model of Parkinson’s disease. Mov. Disord..

[14] Sharma J.C., Bachmann C.G., Linazasoro G. (2010). Classifying risk factors for dyskinesia in Parkinson’s disease. Parkinsonism Relat. Disord..

[15] Putterman D.B., Munhall A.C., Kozell L.B., Belknap J.K., Johnson S.W. (2007). Evaluation of levodopa dose and magnitude of dopamine depletion as risk factors for levodopa-induced dyskinesia in a rat model of Parkinson’s disease. J. Pharmacol. Exp. Ther..

[16] Di Monte D.A., McCormack A., Petzinger G., Janson A.M., Quik M., Langston W.J. (2000). Relationship among nigrostriatal denervation, parkinsonism, and dyskinesias in the MPTP primate model. Mov. Disord..

[17] Schneider J.S., Gonczi H., Decamp E. (2003). Development of levodopa-induced dyskinesias in parkinsonian monkeys may depend upon rate of symptom onset and/or duration of symptoms. Brain Res..

[18] Smith L.A., Jackson M.J., Hansard M.J., Maratos E., Jenner P. (2003). Effect of pulsatile administration of levodopa on dyskinesia induction in drug-naive MPTP-treated common marmosets: Effect of dose, frequency of administration, and brain exposure. Mov. Disord..

[19] Dekker M.C., Bonifati V., van Duijn C.M. (2003). Parkinson’s disease: Piecing together a genetic jigsaw. Brain.

[20] Cai G., Wang H.Y., Friedman E. (2002). Increased dopamine receptor signaling and dopamine receptor-G protein coupling in denervated striatum. J. Pharmacol. Exp. Ther..

[21] Bordet R., Ridray S., Carboni S., Diaz J., Sokoloff P., Schwartz J.C. (1997). Induction of dopamine D3 receptor expression as a mechanism of behavioral sensitization to levodopa. Proc. Natl. Acad. Sci. USA.

[22] Guillin O., Griffon N., Bezard E., Leriche L., Diaz J., Gross C., Sokoloff P. (2003). Brain-derived neurotrophic factor controls dopamine D3 receptor expression: Therapeutic implications in Parkinson’s disease. Eur. J. Pharmacol..

[23] Cote S.R., Kuzhikandathil E.V. (2015). Chronic levodopa treatment alters expression and function of dopamine D3 receptor in the MPTP/p mouse model of Parkinson’s disease. Neurosci. Lett..

[24] Bezard E., Ferry S., Mach U., Stark H., Leriche L., Boraud T., Gross C., Sokoloff P. (2003). Attenuation of levodopa-induced dyskinesia by normalizing dopamine D3 receptor function. Nat. Med..

[25] Azkona G., Sagarduy A., Aristieta A., Vazquez N., Zubillaga V., Ruiz-Ortega J.A., Perez-Navarro E., Ugedo L., Sanchez-Pernaute R. (2014). Buspirone anti-dyskinetic effect is correlated with temporal normalization of dysregulated striatal DRD1 signalling in L-DOPA-treated rats. Neuropharmacology.

[26] Cote S.R., Chitravanshi V.C., Bleickardt C., Sapru H.N., Kuzhikandathil E.V. (2014). Overexpression of the dopamine D3 receptor in the rat dorsal striatum induces dyskinetic behaviors. Behav. Brain Res..

[27] Marcellino D., Ferre S., Casado V., Cortes A., Le Foll B., Mazzola C., Drago F., Saur O., Stark H., Soriano A. (2008). Identification of dopamine D1-D3 receptor heteromers. Indications for a role of synergistic D1-D3 receptor interactions in the striatum. J. Biol. Chem..

[28] Fiorentini C., Busi C., Gorruso E., Gotti C., Spano P., Missale C. (2008). Reciprocal regulation of dopamine D1 and D3 receptor function and trafficking by heterodimerization. Mol. Pharmacol..

[29] Cruz-Trujillo R., Avalos-Fuentes A., Rangel-Barajas C., Paz-Bermudez F., Sierra A., Escartin-Perez E., Aceves J., Erlij D., Floran B. (2013). D3 dopamine receptors interact with dopamine D1 but not D4 receptors in the GABAergic terminals of the SNr of the rat. Neuropharmacology.

[30] Albarran S., Paz-Bermudez F., Erlij D., Aceves J., Florán B. (2013). Dopamine D3 receptor prevents stimulation of [3H] GABA release in substantia nigra pars reticulata of hemiparkinsonian dyskinetic rats. Soc. Neurosc. Abstr..

[31] Avalos-Fuentes A., Albarran-Bravo S., Loya-Lopez S., Cortes H., Recillas-Morales S., Magana J.J., Paz-Bermudez F., Rangel-Barajas C., Aceves J., Erlij D. (2015). Dopaminergic denervation switches dopamine D3 receptor signaling and disrupts its Ca(2+) dependent modulation by CaMKII and calmodulin in striatonigral projections of the rat. Neurobiol. Dis..

[32] Hudson J.L., van Horne C.G., Stromberg I., Brock S., Clayton J., Masserano J., Hoffer B.J., Gerhardt G.A. (1993). Correlation of apomorphine- and amphetamine-induced turning with nigrostriatal dopamine content in unilateral 6-hydroxydopamine lesioned rats. Brain Res..

[33] Nash J.E., Johnston T.H., Collingridge G.L., Garner C.C., Brotchie J.M. (2005). Subcellular redistribution of the synapse-associated proteins PSD-95 and SAP97 in animal models of Parkinson’s disease and L-DOPA-induced dyskinesia. FASEB J..

[34] Mela F., Marti M., Dekundy A., Danysz W., Morari M., Cenci M.A. (2007). Antagonism of metabotropic glutamate receptor type 5 attenuates l-DOPA-induced dyskinesia and its molecular and neurochemical correlates in a rat model of Parkinson’s disease. J. Neurochem..

[35] Hagell P., Widner H. (1999). Clinical rating of dyskinesias in Parkinson’s disease: Use and reliability of a new rating scale. Mov. Disord..

[36] Lundblad M., Andersson M., Winkler C., Kirik D., Wierup N., Cenci M.A. (2002). Pharmacological validation of behavioural measures of akinesia and dyskinesia in a rat model of Parkinson’s disease. Eur. J. Neurosci..

[37] Schallert T., Fleming S.M., Leasure J.L., Tillerson J.L., Bland S.T. (2000). CNS plasticity and assessment of forelimb sensorimotor outcome in unilateral rat models of stroke, cortical ablation, parkinsonism and spinal cord injury. Neuropharmacology.

[38] Alexander S.P. (1995). The measurement of cyclic AMP levels in biological preparations. Methods Mol. Biol..

[39] Nava-Asbell C., Paz-Bermudez F., Erlij D., Aceves J., Floran B. (2007). GABA(B) receptor activation inhibits dopamine D1 receptor-mediated facilitation of [(3)H]GABA release in substantia nigra pars reticulata. Neuropharmacology.

[40] Ungerstedt U., Arbuthnott G.W. (1970). Quantitative recording of rotational behavior in rats after 6-hydroxy-dopamine lesions of the nigrostriatal dopamine system. Brain Res..

[41] Avalos-Fuentes A., Loya-Lopez S., Flores-Perez A., Recillas-Morales S., Cortes H., Paz-Bermudez F., Aceves J., Erlij D., Floran B. (2013). Presynaptic CaMKIIalpha modulates dopamine D3 receptor activation in striatonigral terminals of the rat brain in a Ca(2)(+) dependent manner. Neuropharmacology.

[42] Hefti F., Melamed E., Sahakian B.J., Wurtman R.J. (1980). Circling behavior in rats with partial, unilateral nigro-striatal lesions: Effect of amphetamine, apomorphine, and DOPA. Pharmacol. Biochem. Behav..

[43] Boix J., Padel T., Paul G. (2015). A partial lesion model of Parkinson’s disease in mice—Characterization of a 6-OHDA-induced medial forebrain bundle lesion. Behav. Brain Res..

[44] Iancu R., Mohapel P., Brundin P., Paul G. (2005). Behavioral characterization of a unilateral 6-OHDA-lesion model of Parkinson’s disease in mice. Behav. Brain Res..

[45] Przedborski S., Levivier M., Jiang H., Ferreira M., Jackson-Lewis V., Donaldson D., Togasaki D.M. (1995). Dose-dependent lesions of the dopaminergic nigrostriatal pathway induced by intrastriatal injection of 6-hydroxydopamine. Neuroscience.

[46] Ahlskog J.E., Muenter M.D. (2001). Frequency of levodopa-related dyskinesias and motor fluctuations as estimated from the cumulative literature. Mov. Disord..

[47] O’Sullivan S.S., Williams D.R., Gallagher D.A., Massey L.A., Silveira-Moriyama L., Lees A.J. (2008). Nonmotor symptoms as presenting complaints in Parkinson’s disease: A clinicopathological study. Mov. Disord..

[48] Nadjar A., Gerfen C.R., Bezard E. (2009). Priming for l-dopa-induced dyskinesia in Parkinson’s disease: A feature inherent to the treatment or the disease?. Prog. Neurobiol..

[49] dos Santos E.U.D., Duarte E.B.C., Miranda L.M.R., Asano A.G.C., Asano N.M.J., Maia M.M.D., de Souza P.R.E. (2019). Influence of DRD1 and DRD3 Polymorphisms in the Occurrence of Motor Effects in Patients with Sporadic Parkinson’s Disease. NeuroMolecular Med..

[50] Martín-Flores N., Fernández-Santiago R., Antonelli F., Cerquera C., Moreno V., Martí M.J., Ezquerra M., Malagelada C. (2019). MTOR Pathway-Based Discovery of Genetic Susceptibility to L-DOPA-Induced Dyskinesia in Parkinson’s Disease Patients. Mol. Neurobiol..

[51] Cerasa A., Salsone M., Morelli M., Pugliese P., Arabia G., Gioia C.M., Novellino F., Quattrone A. (2013). Age at onset influences neurodegenerative processes underlying PD with levodopa-induced dyskinesias. Parkinsonism Relat. Disord..

[52] Luquin M.R., Scipioni O., Vaamonde J., Gershanik O., Obeso J.A. (1992). Levodopa-induced dyskinesias in Parkinson’s disease: Clinical and pharmacological classification. Mov. Disord..

[53] Fox S.H., Lang A.E. (2008). Levodopa-related motor complications--phenomenology. Mov. Disord..

[54] Goubault E., Nguyen H.P., Bogard S., Blanchet P.J., Bezard E., Vincent C., Langlois M., Duval C. (2018). Cardinal Motor Features of Parkinson’s Disease Coexist with Peak-Dose Choreic-Type Drug-Induced Dyskinesia. J. Parkinsons Dis..

[55] Papa S.M., Engber T.M., Kask A.M., Chase T.N. (1994). Motor fluctuations in levodopa treated parkinsonian rats: Relation to lesion extent and treatment duration. Brain Res..

[56] Giorgi M., D’Angelo V., Esposito Z., Nuccetelli V., Sorge R., Martorana A., Stefani A., Bernardi G., Sancesario G. (2008). Lowered cAMP and cGMP signalling in the brain during levodopa-induced dyskinesias in hemiparkinsonian rats: New aspects in the pathogenetic mechanisms. Eur. J. Neurosci..

[57] Picconi B., Centonze D., Hakansson K., Bernardi G., Greengard P., Fisone G., Cenci M.A., Calabresi P. (2003). Loss of bidirectional striatal synaptic plasticity in L-DOPA-induced dyskinesia. Nat. Neurosci..

[58] Feyder M., Bonito-Oliva A., Fisone G. (2011). L-DOPA-Induced Dyskinesia and Abnormal Signaling in Striatal Medium Spiny Neurons: Focus on Dopamine D1 Receptor-Mediated Transmission. Front. Behav. Neurosci..

[59] Fisone G., Bezard E. (2011). Molecular mechanisms of l-DOPA-induced dyskinesia. Int. Rev. Neurobiol..

[60] Guitart X., Navarro G., Moreno E., Yano H., Cai N.S., Sanchez-Soto M., Kumar-Barodia S., Naidu Y.T., Mallol J., Cortes A. (2014). Functional selectivity of allosteric interactions within G protein-coupled receptor oligomers: The dopamine D1-D3 receptor heterotetramer. Mol. Pharmacol..

[61] Solis O., Moratalla R. (2018). Dopamine receptors: Homomeric and heteromeric complexes in L-DOPA-induced dyskinesia. J. Neural Transm..

[62] Griffon N., Pilon C., Sautel F., Schwartz J.C., Sokoloff P. (1997). Two intracellular signaling pathways for the dopamine D3 receptor: Opposite and synergistic interactions with cyclic AMP. J. Neurochem..

[63] Schwartz J.C., Diaz J., Bordet R., Griffon N., Perachon S., Pilon C., Ridray S., Sokoloff P. (1998). Functional implications of multiple dopamine receptor subtypes: The D1/D3 receptor coexistence. Brain Res. Rev..

[64] Albin R.L., Young A.B., Penney J.B. (1989). The functional anatomy of basal ganglia disorders. Trends Neurosci..

[65] DeLong M.R. (1990). Primate models of movement disorders of basal ganglia origin. Trends Neurosci..

[66] Rangel-Barajas C., Silva I., Garcia-Ramirez M., Sanchez-Lemus E., Floran L., Aceves J., Erlij D., Floran B. (2008). 6-OHDA-induced hemiparkinsonism and chronic L-DOPA treatment increase dopamine D1-stimulated [(3)H]-GABA release and [(3)H]-cAMP production in substantia nigra pars reticulata of the rat. Neuropharmacology.

[67] Mela F., Marti M., Bido S., Cenci M.A., Morari M. (2012). In vivo evidence for a differential contribution of striatal and nigral D1 and D2 receptors to L-DOPA induced dyskinesia and the accompanying surge of nigral amino acid levels. Neurobiol. Dis..

[68] Deniau J.M., Mailly P., Maurice N., Charpier S. (2007). The pars reticulata of the substantia nigra: A window to basal ganglia output. Prog. Brain Res..

[69] Windels F., Kiyatkin E.A. (2004). GABA, not glutamate, controls the activity of substantia nigra reticulata neurons in awake, unrestrained rats. J. Neurosci..

[70] Kumar R., Riddle L.R., Griffin S.A., Chu W., Vangveravong S., Neisewander J., Mach R.H., Luedtke R.R. (2009). Evaluation of D2 and D3 dopamine receptor selective compounds on L-dopa-dependent abnormal involuntary movements in rats. Neuropharmacology.

[71] Riddle L.R., Kumar R., Griffin S.A., Grundt P., Newman A.H., Luedtke R.R. (2011). Evaluation of the D3 dopamine receptor selective agonist/partial agonist PG01042 on L-dopa dependent animal involuntary movements in rats. Neuropharmacology.

[72] Kumar N., Van Gerpen J.A., Bower J.H., Ahlskog J.E. (2005). Levodopa-dyskinesia incidence by age of Parkinson’s disease onset. Mov. Disord..

[73] Kumar R., Riddle L., Griffin S.A., Grundt P., Newman A.H., Luedtke R.R. (2009). Evaluation of the D3 dopamine receptor selective antagonist PG01037 on L-dopa-dependent abnormal involuntary movements in rats. Neuropharmacology.

[74] Visanji N.P., Fox S.H., Johnston T., Reyes G., Millan M.J., Brotchie J.M. (2009). Dopamine D3 receptor stimulation underlies the development of L-DOPA-induced dyskinesia in animal models of Parkinson’s disease. Neurobiol. Dis..

[75] Solis O., Garcia-Montes J.R., Gonzalez-Granillo A., Xu M., Moratalla R. (2015). Dopamine D3 Receptor Modulates l-DOPA-Induced Dyskinesia by Targeting D1 Receptor-Mediated Striatal Signaling. Cereb. Cortex..

[76] Mela F., Millan M.J., Brocco M., Morari M. (2010). The selective D(3) receptor antagonist, S33084, improves parkinsonian-like motor dysfunction but does not affect L-DOPA-induced dyskinesia in 6-hydroxydopamine hemi-lesioned rats. Neuropharmacology.

[77] Flores G., Liang J.J., Sierra A., Martinez-Fong D., Quirion R., Aceves J., Srivastava L.K. (1999). Expression of dopamine receptors in the subthalamic nucleus of the rat: Characterization using reverse transcriptase-polymerase chain reaction and autoradiography. Neuroscience.

[78] Briones-Lizardi L.J., Cortes H., Avalos-Fuentes J.A., Paz-Bermudez F.J., Aceves J., Erlij D., Floran B. (2019). Presynaptic control of [(3)H]-glutamate release by dopamine receptor subtypes in the rat substantia nigra. Central role of D1 and D3 receptors. Neuroscience.

[79] Ibanez-Sandoval O., Hernandez A., Floran B., Galarraga E., Tapia D., Valdiosera R., Erlij D., Aceves J., Bargas J. (2006). Control of the subthalamic innervation of substantia nigra pars reticulata by D1 and D2 dopamine receptors. J. Neurophysiol..

[80] Shen K.Z., Johnson S.W. (2012). Regulation of polysynaptic subthalamonigral transmission by D2, D3 and D4 dopamine receptors in rat brain slices. J. Physiol..

[81] Hubert G.W., Paquet M., Smith Y. (2001). Differential subcellular localization of mGluR1a and mGluR5 in the rat and monkey Substantia nigra. J. Neurosci..

[82] Romano C., Sesma M.A., McDonald C.T., O’Malley K., Van den Pol A.N., Olney J.W. (1995). Distribution of metabotropic glutamate receptor mGluR5 immunoreactivity in rat brain. J. Comp. Neurol..

[83] Tallaksen-Greene S.J., Kaatz K.W., Romano C., Albin R.L. (1998). Localization of mGluR1a-like immunoreactivity and mGluR5-like immunoreactivity in identified populations of striatal neurons. Brain Res..

[84] van den Pol A.N., Romano C., Ghosh P. (1995). Metabotropic glutamate receptor mGluR5 subcellular distribution and developmental expression in hypothalamus. J. Comp. Neurol..

[85] Wang J., Johnson K.M. (1995). Regulation of striatal cyclic-3’,5’-adenosine monophosphate accumulation and GABA release by glutamate metabotropic and dopamine D1 receptors. J. Pharmacol. Exp. Ther..

[86] Garcia-Montes J.R., Solis O., Enriquez-Traba J., Ruiz-DeDiego I., Drucker-Colin R., Moratalla R. (2018). Genetic Knockdown of mGluR5 in Striatal D1R-Containing Neurons Attenuates L-DOPA-Induced Dyskinesia in Aphakia Mice. Mol. Neurobiol..

[87] Lanza K., Meadows S.M., Chambers N.E., Nuss E., Deak M.M., Ferre S., Bishop C. (2018). Behavioral and cellular dopamine D1 and D3 receptor-mediated synergy: Implications for L-DOPA-induced dyskinesia. Neuropharmacology.

[88] Farre D., Munoz A., Moreno E., Reyes-Resina I., Canet-Pons J., Dopeso-Reyes I.G., Rico A.J., Lluis C., Mallol J., Navarro G. (2015). Stronger Dopamine D1 Receptor-Mediated Neurotransmission in Dyskinesia. Mol. Neurobiol..

